# Supplementary material for: Bio-inspired magnetic-driven folded diaphragm for biomimetic robot
Source: Nat Commun. 2023 Jan 11;14:163. doi: 10.1038/s41467-023-35905-6 (PMC9834404; doi:10.1038/s41467-023-35905-6)
Supplement: Supplementary file 1 — Supplementary Information [file 41467_2023_35905_MOESM1_ESM.pdf]

# Supplementary information

# Bio-inspired magnetic-driven folded diaphragm for biomimetic robot

Dezhao Lin [1], Fan Yang[1]✉, Di Gong [1,2], Ruihong Li [1]

[1] Research Center for Intelligent Materials and Structures (CIMS), College of Mechanical Engineering and Automation, Huaqiao University, Xiamen, Fujian, P.R.China

[2] Institute of Extremely-Weak-Magnetic-Field Massive Scientific Instrumentation Facility, Hangzhou, Zhejiang, P. R.China

✉E-mail: xmyf@hotmail.com.

## Supplementary Note 1. The deformation mechanism of folded diaphragm

Due to the folded structure of the circular diaphragm shown in Supplementary Figure 1a, the deformation mechanism of magnetic diaphragm in each segment can be assumed (simplified) as a multi-link mechanism with rotation joint ($J_i$) located at each segment end point, as shown in Supplementary Figure 1b, and the individual segment can be considered as ideally stiff, and connected by torsion spring at the edge. In this example, the multi-link mechanism, consisting of 5 connecting rods ($b_1$~$b_5$) and 5 rotation joints ($J_1$~$J_5$), which is driven by the magnetic driving torque ($\tau_m$) and elastic-resistance torque ($\tau_e$) shown in Supplementary Figure 1b together with the initial joint angle equal to $\pi/2$, as shown in Supplementary Figure 1c. Parameters $\alpha_1$~$\alpha_4$ in Supplementary Figure 1b represent the angle between the direction of the magnetic moment ($\vec{m_i}$) of each segment and the direction of the active magnetic field ($\vec{B}$).

Considering the above definition, the magnetic torque ($\tau_m$) shown in Supplementary Figure 1b generated by the magnetically connected rods in unit radius can be evaluated based on[1]:

$$\tau_m = \int_0^{2\pi} \sum_{i=1}^{4} \left[N_i\left(\vec{m_i} \times \vec{B}\right)\right] d\varphi = \int_0^{2\pi} \sum_{i=1}^{4} \left(N_i|\vec{m_i}|\left|\vec{B}\right|sin(\alpha_i)\right) d\varphi \tag{1}$$

where $\varphi$ represents the angle of cylindrical coordinates shown in Supplementary Figure 1a, and $N_i$ is the number of magnetically micro-particles in each connecting rod; the elastic-resistance torque ($\tau_e$) of the rotating joint can be expressed as[2]:

$$\tau_e = \int_0^{2\pi} \sum_{i=1}^{5} (-k_i \cdot |\Delta\theta_i|)\, d\varphi \tag{2}$$

where $k_i$ is the mechanical stiffness, which is the function of elastic modulus and moment inertia[2]. Here, $\Delta\theta_i$ $(i = 1{\sim}5)$ represent the rotation angle around the rotating joints ($J_i$).

Based on the assumption of ideally stiffness of the individual segment and rotation joint ($J_i$) between each segment, the final deformation of the folded diaphragm can be evaluated based on the toque balance between the magnetic driving torque ($\tau_m$) and elastic-resistance torque ($\tau_e$), as：

$$\tau_m + \tau_e = 0 \tag{3}$$

Besides, the geometrical constrain based on the assumption of the constant total area of the folded diaphragm can be expressed as:

$$\sum_{i=1}^{4} |l_i cos\beta_i| = \text{constant} \tag{4}$$

where $l_i$ is length of each segment; the constant is equal to 7mm in this case, as shown in Supplementary Figure 1a.

For example, under the condition of vertical upward magnetic field, considering the ideal magnetization conditions, the initial angles between the magnetization direction of magnetic micro-particles and the magnetic field are $7\pi/18$ in the connected rods 1 and 3 and $8\pi/9$ in the connected rods 2 and 4. Therefore, $\alpha_1$~$\alpha_4$ can be expressed as:

$$\begin{cases} \alpha_1 = \frac{7\pi}{18} - \Delta\theta_1 \\ \alpha_2 = \frac{8\pi}{9} - \Delta\theta_1 - \Delta\theta_2 \\ \alpha_3 = \frac{7\pi}{18} - \Delta\theta_1 - \Delta\theta_2 + \Delta\theta_3 \\ \alpha_4 = \frac{8\pi}{9} - \Delta\theta_1 - \Delta\theta_2 + \Delta\theta_3 - \Delta\theta_4 \end{cases} \tag{5}$$

The maximum rotation angle of the connected rod is the magnetization direction parallel with the magnetic field direction, so the connected rod 2 and 4 can be rotated by $8\pi/9$ while the connected rod 1 and 3 can be rotated by $7\pi/18$. Therefore, under the vertical upward magnetic field, the connected rod 2 and 4 are the main deformation segment and then generate the upward displacement of central vertex.

Under the condition of vertical downward magnetic field, considering the ideal magnetization conditions, the initial angles between the magnetization direction of magnetic micro-particles and the magnetic field are $-11\pi/18$ in connected rods 1 and 3 and $-\pi/9$ in connected rods 2 and 4, respectively. Therefore, $\alpha_1$~$\alpha_4$ can be expressed as:

$$\begin{cases} \alpha_1 = -\frac{11\pi}{18} - \Delta\theta_1 \\ \alpha_2 = -\frac{\pi}{9} - \Delta\theta_1 - \Delta\theta_2 \\ \alpha_3 = -\frac{11\pi}{18} - \Delta\theta_1 - \Delta\theta_2 + \Delta\theta_3 \\ \alpha_4 = -\frac{\pi}{9} - \Delta\theta_1 - \Delta\theta_2 + \Delta\theta_3 - \Delta\theta_4 \end{cases} \tag{6}$$

It can be found from Supplementary Equations (5) ~ (6) that under the vertical downward magnetic field, the connected rod 1 and 3 are main deformation segment, because the connected rod 1 and 3 can be rotated by $11\pi/18$ while the connected rod 2 and 3 can be rotated by $\pi/9$, and then generate the downward displacement of central vertex.

Considering the above, assuming the fifth connection rod ($b_5$ in Supplementary Figure 1b) is the free end, the angle of each connection rod related to the horizontal line, $\beta_1$~$\beta_5$ shown in Supplementary Figure 1c, can be expressed as:

$$\begin{cases} \beta_1 = \frac{\pi}{4} + \Delta\theta_1 \\ \beta_2 = \pi - \frac{\pi}{2} - \Delta\theta_2 - \beta_1 \\ \beta_3 = \pi - \frac{\pi}{2} - \Delta\theta_3 - \beta_2 \\ \beta_4 = \pi - \frac{\pi}{2} - \Delta\theta_4 - \beta_3 \\ \Delta\theta_5 = \pi - \frac{\pi}{2} - \frac{\pi}{4} - \beta_4 \end{cases} \tag{7}$$

Base on Supplementary Figure 1c, Supplementary Equation (7) can be rearranged as:

$$\begin{cases} \beta_1 = \frac{\pi}{4} + \Delta\theta_1 \\ \beta_2 = \frac{\pi}{4} - \Delta\theta_1 - \Delta\theta_2 \\ \beta_3 = \frac{\pi}{4} + \Delta\theta_1 + \Delta\theta_2 - \Delta\theta_3 \\ \beta_4 = \frac{\pi}{4} - \Delta\theta_1 - \Delta\theta_2 + \Delta\theta_3 - \Delta\theta_4 \\ \Delta\theta_5 = \Delta\theta_1 + \Delta\theta_2 - \Delta\theta_3 + \Delta\theta_4 \end{cases} \tag{8}$$

Substituting Supplementary Equations (5) ~ (8) to Supplementary Equation (4), one can obtain the final deformation of the top point of the central vertex, as shown in Supplementary Figure 1a as:

$$Dis = \sum_{i=1}^{4} \left| l_i \left[ sin\beta_i - \sin\left(\frac{\pi}{4}\right) \right] \right| \tag{9}$$

## Supplementary Note 2. Simulations of the folded diaphragm and its applications

The deformations of the proposed magnetic-driven folded diaphragm and its applications are simulated by COMSOL Multiphysics 5.4 (COMSOL Inc., Sweden). Since the circular folded diaphragm is a symmetrical structure, as illustrated in Supplementary Figure 1, in this study, to facilitate and expedite the simulation procedure, the section of circular folded diaphragm (or section of soft robot/actuator) with 0.1 mm thickness, as illustrated in Supplementary Figure 2, has been utilized to establish the multi-physics Finite Element Method (FEM) model, which also includes the magnetic sheet to achieve the single direction action purpose, as illustrated in Supplementary Figure 2c-e. The models are meshed though the function of Physics-Controlled Mesh with Extremely fine mesh, which automatically split the mesh based on the settings of the physics dimension and boundaries in the model subjected to 40 mT driving field (upward direction) generated by permanent magnets.

In the simulation model, the material of the folded diaphragm and magnetic sheet are set as linear elastic, whose effective density, effective Young's modulus and Poisson's ratio are 1.86 g cm^-3, 84.5 kPa, and 0.49, respectively[3]. Each segment is connected by the boundary of the flexible attachment. The magnetization magnitudes of the magnetic materials[3] are set as 62000 A m^-1, whose components on the X-axis and Y axis are calculated through the ideal magnetization angle shown in Fig. 1d, Fig. 3a, e, Fig. 4a, and Fig. 5d. The deformations of the proposed magnetic-driven folded diaphragm and its applications are analyzed by the interactive calculation of magnetic field module and the solid mechanics module. The comparisons between the experiments and simulation results are shown in Supplementary Figure 2.

## Supplementary Note 3. The deformation properties of the folded diaphragm

The deformation performance of the proposed folded diaphragm depends on the elastic-resistance force, magnetic driving force and the length/height of each segment, as presented in Supplementary Note 1. One can easily realize that the above three factors are all related to the diaphragm properties during the fabrication procedure, which include the diaphragm thickness, folding degree, number of segments, hardness of the silicone elastomer, weight ratio of NdFeB particles to silicone elastomer and the magnetization degree. Here, 14 type of circle diaphragm subjected to different fabrication conditions, as summarized in Supplementary Table 1, are prepared to investigate the deformation properties of the folded diaphragm.

Diaphragm Thickness: Supplementary Figure 3 shows deformation performance of the diaphragms with different thickness (0.5 mm, 1.0 mm, 1.5 mm, Sample 1 to 3 in Supplementary Table 1). It can be found from Supplementary Figure 3 that the elastic-resistance force increases significantly with the increase of diaphragm thickness, and then decrease the displacement of the diaphragm although the number of magnetic particles also increases with the increase volume of the diaphragm, which will also increase the magnetic driving force.

Diaphragm Folded Degree: Supplementary Figure 4 shows the deformation performance of the diaphragms with different folded degrees (35°, 45°, 55°, Sample 4, 1 and 5 in Supplementary Table 1). Different folded degrees result in different segment lengths (2.44 mm, 2.83 mm, 3.49 mm corresponding to 35°, 45°, 55°) and diaphragm heights (1.40 mm, 2.00 mm, 2.85 mm), as shown in Supplementary Figure 4. Compared with the diaphragm with 45° folded degree, the diaphragm with 35° folded degree with the smaller segment length, which limits the deformation of the diaphragm together with the larger elastic-resistance force, so the deformation of it is smaller than the diaphragm with 45° folded degree. Compared with the diaphragm with 45° folded degree, the segments of the diaphragm with 55° folded degree may reach the balance position quickly with 65° magnetization degree, which can be analyzed through Supplementary Equations (1) ~ (9). Therefore, for the folded diaphragm with the larger folded degree, the larger magnetization degree should be applied to amplify the deformation of the folded diaphragm.

Number of the segments: Supplementary Figure 5 shows the deformation performance of the diaphragms with different numbers of the segment (3, 5, 6, 7, Sample 6, 1, 7 and 8 in Supplementary Table 1). Due to the large center segment, which cannot achieve deformation, the deformation of the diaphragm with 3 segments is smaller than the diaphragm with 5 segments. Due to the larger elastic-resistance caused by the much more folded segments, the deformations of the diaphragm with 6 and 9 segments is smaller than the diaphragm with 5 segments, too.

Diaphragm Hardness: Supplementary Figure 6 shows the deformation performance of the diaphragms with different hardness of the silicon rubber, including Ecoflex-10 (Shore Hardness: 00-10), Ecoflex-20 (Shore Hardness: 00-20), Ecoflex-50 (Shore Hardness: 00-50) associated with Sample 9, 1 and 10 in Supplementary Table 1. It can be found from Supplementary Figure 6, the increase of the hardness of the silicon rubber causes the increase of the elastic-resistance force, and then decreases the deformation of the folded diaphragm.

Diaphragm Weight Ratio: Supplementary Figure 7 shows the deformation performance of the

diaphragms with different weight ratios of NdFeB particles to silicone elastomer (2:3, 1:1, 3:2, Sample 11, 1 and 12 in Supplementary Table 1). Although the elastic-resistance force increases due to the increase of weight ratio, the increase of magnetic force caused by the increase number of NdFeB particles make the diaphragm deformation slightly increase.

Diaphragm magnetization direction: Supplementary Figure 8 shows the deformation performance of the diaphragms with different magnetization degrees (45°, 55°, 65°, Sample 13, 1 and 14 in Supplementary Table 1). It can be found that the deformation of the diaphragm increases by the increase of the magnetization angle, which is due to the fact that the larger magnetization angle results in the larger magnetic torque, as shown in Supplementary Equation (2).

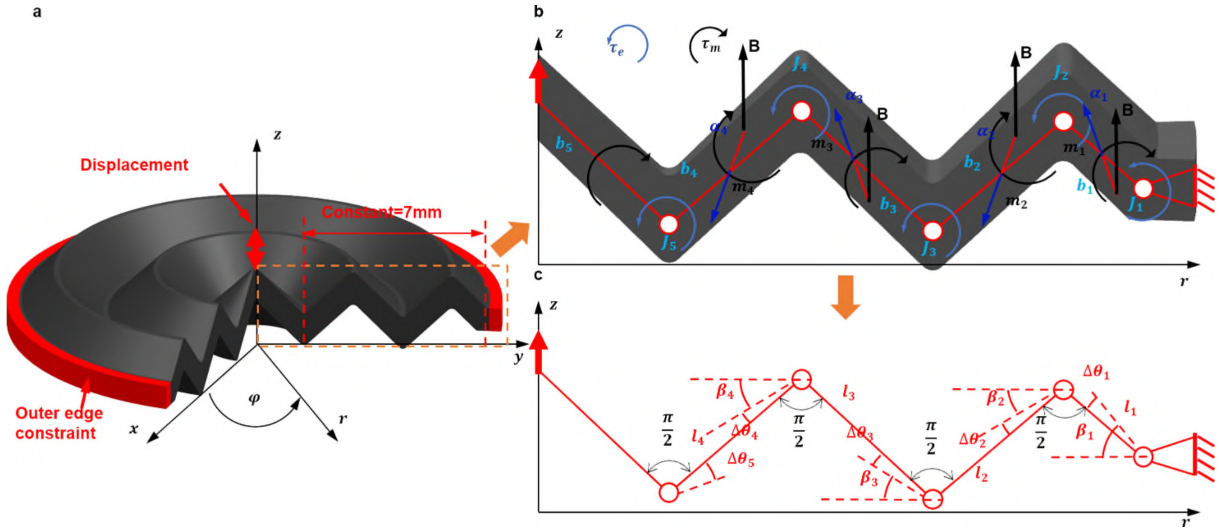


Supplementary Figure 1. **The schematic of deformation mechanism of folded diaphragm. a** The folded structure of the circular diaphragm. **b** The deformation mechanism. **c** The simplified multi-link mechanism.

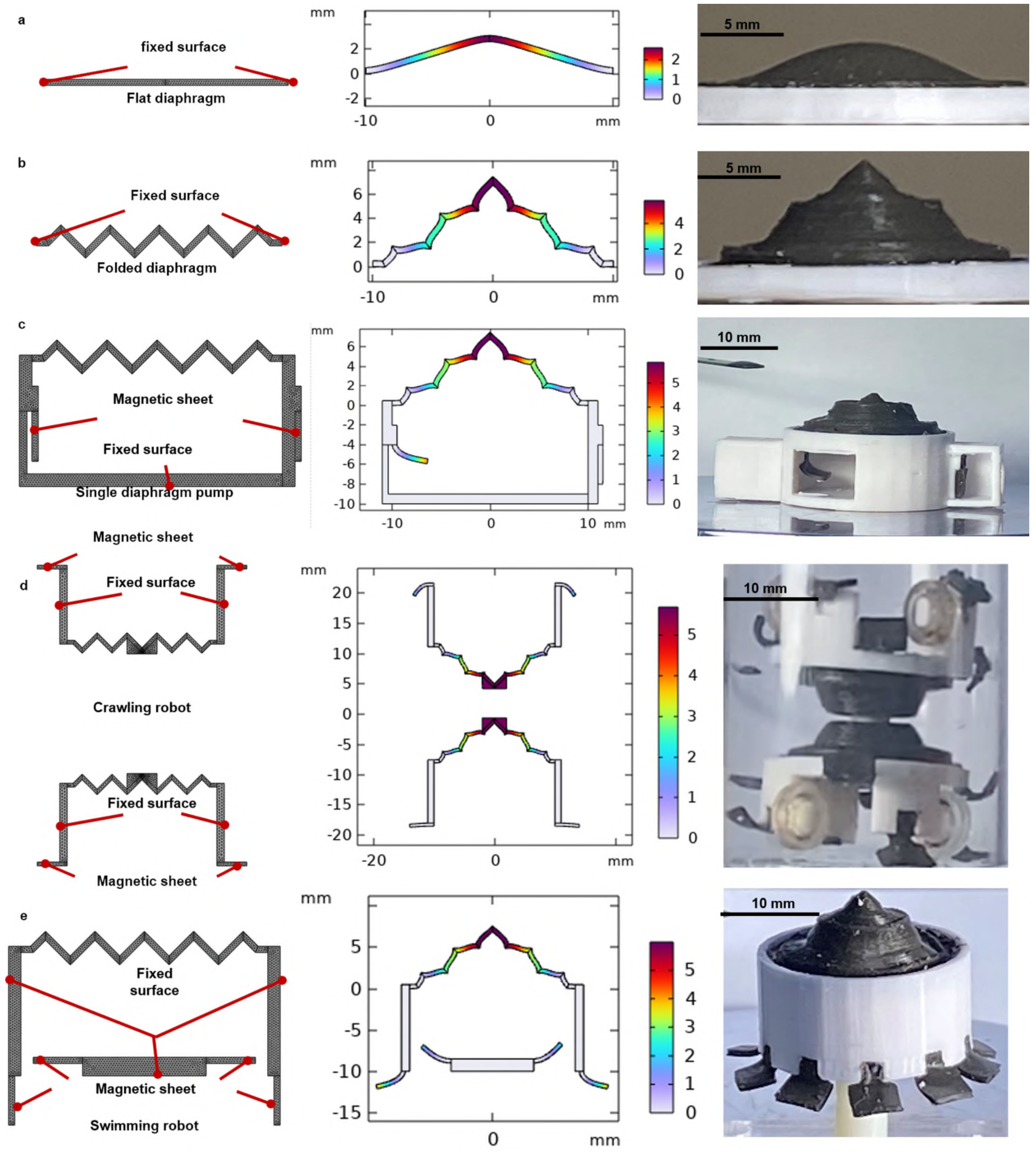


Supplementary Figure 2. **Simulations and results comparison of the folded diaphragm and its applications. a** Flat diaphragm. **b** Folded diaphragm. **c** Single diaphragm pump. **d** Crawling robot. **e** Swimming robot.

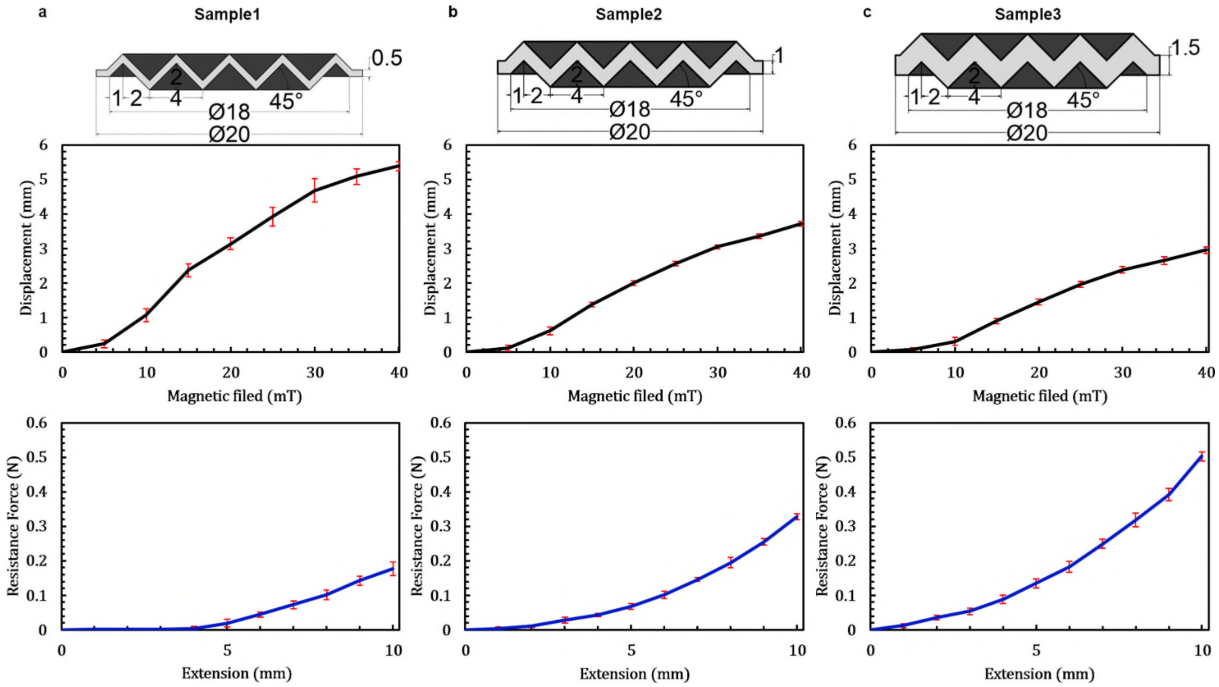


Supplementary Figure 3. **The deformation and elastic-resistance force of the folded diaphragm (**average value and standard deviation of 9 cycles measurement**) with different thicknesses**. **a** 0.5 mm (Sample 1). **b** 1.0 mm (Sample 2). **c** 1.5mm (Sample 3). Detailed information of different samples has been summarized in Supplementary Table 1.

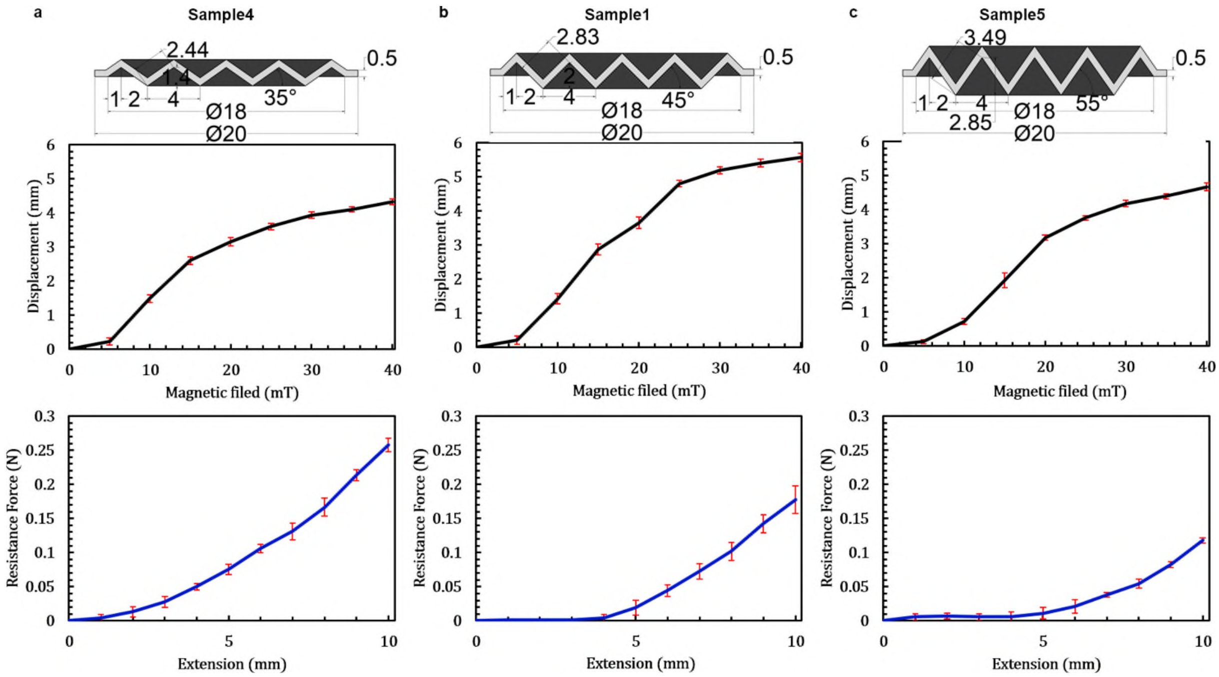


Supplementary Figure 4. **The deformation and elastic-resistance force of the folded diaphragm (**average value and standard deviation of 9 cycles measurement**) with different folding degrees**. **a** 35° (Sample 4). **b** 45° (Sample 1). **c** 55° (Sample 5). Detailed information of different samples has been summarized in Supplementary Table 1.

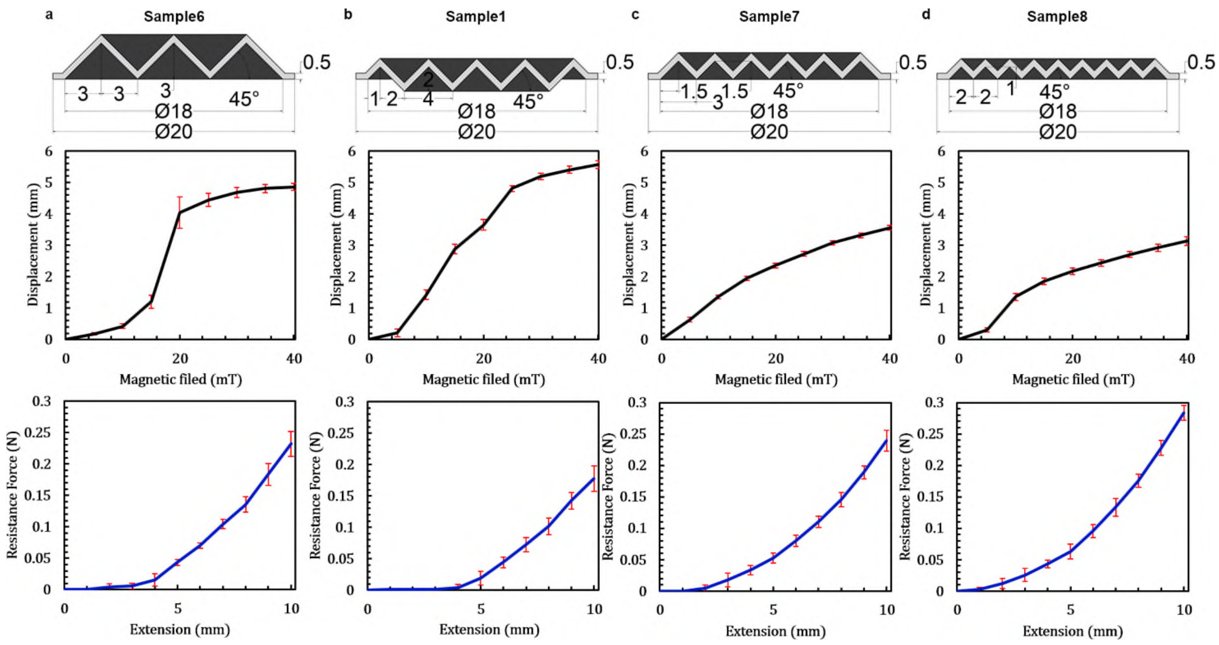


Supplementary Figure 5. **The deformation and elastic-resistance force of the folded diaphragm (**average value and standard deviation of 9 cycles measurement**) with different segment numbers. a** 3 (Sample 6). **b** 5 (Sample 1). **c** 6 (Sample 7). **d** 9 (Sample 8). Detailed information of different samples has been summarized in Supplementary Table 1.

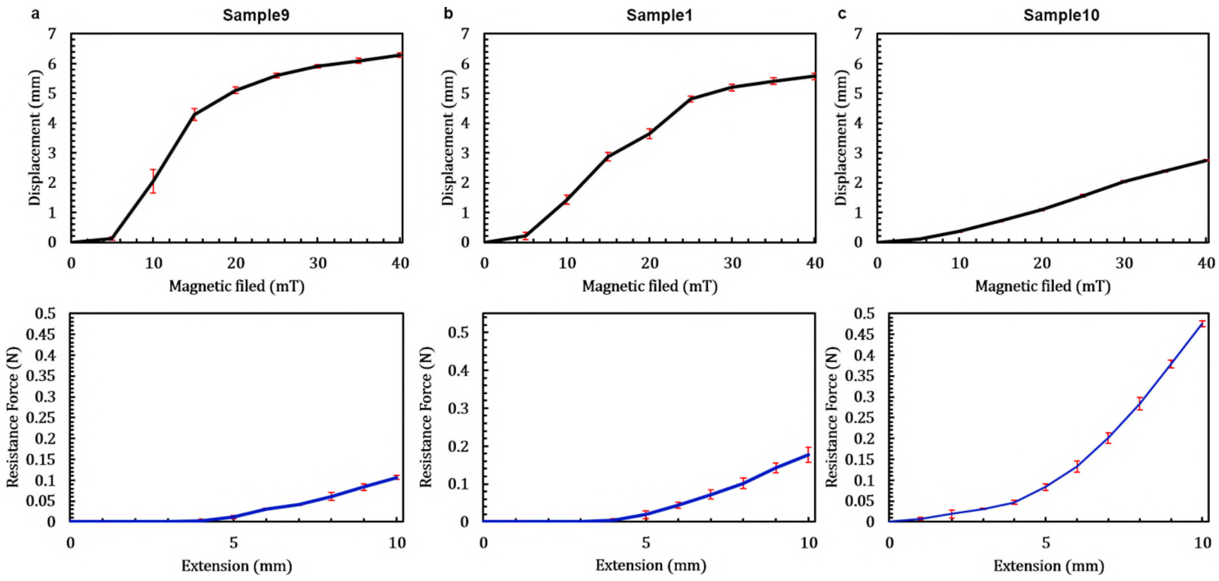


Supplementary Figure 6. **The deformation and elastic-resistance force of the folded diaphragm (**average value and standard deviation of 9 cycles measurement**) with different hardness of the silicon rubber**. **a** Ecoflex-10 (Sample 9). **b** Ecoflex-20 (Sample 1). **c** Ecoflex-50 (Sample 10). Detailed information of different samples has been summarized in Supplementary Table 1.

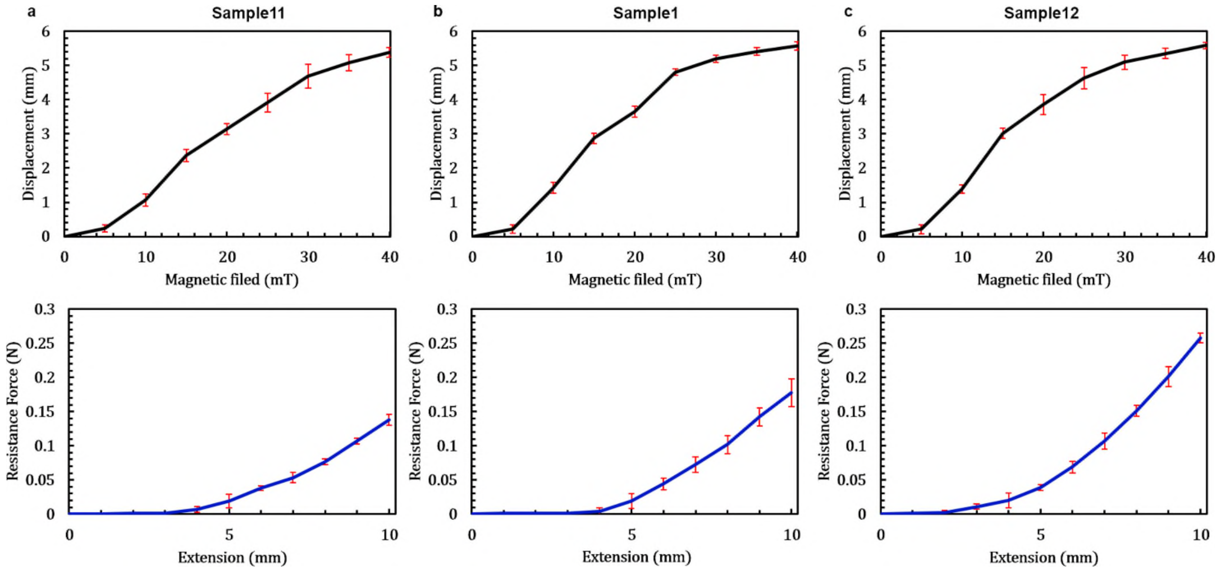


Supplementary Figure 7. **The deformation and elastic-resistance force of the folded diaphragm (**average value and standard deviation of 9 cycles measurement**) with different weight ratios of NdFeB particles to silicone elastomer. a** 2:3 (Sample 11). **b** 1:1(Sample 1). **c** 3:2 (Sample 12). Detailed information of different samples has been summarized in Supplementary Table 1.

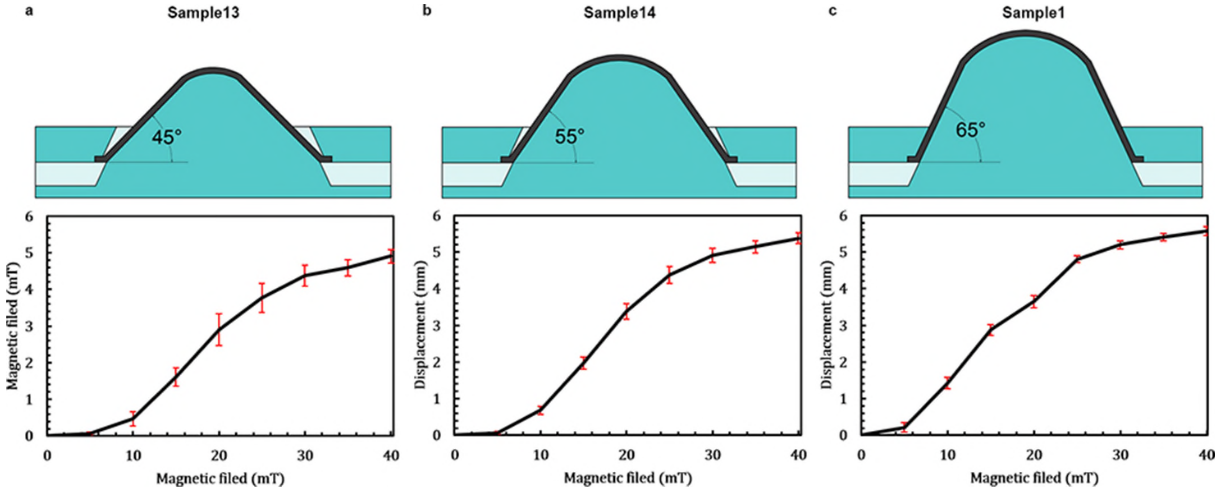


Supplementary Figure 8. **The deformation of the folded diaphragm (**average value and standard deviation of 9 cycles measurement**) with different magnetization degrees. a** 45° (Sample 13). **b** 55° (Sample 1). **c** 65° (Sample 14). Detailed information of different samples has been summarized in Supplementary Table 1.

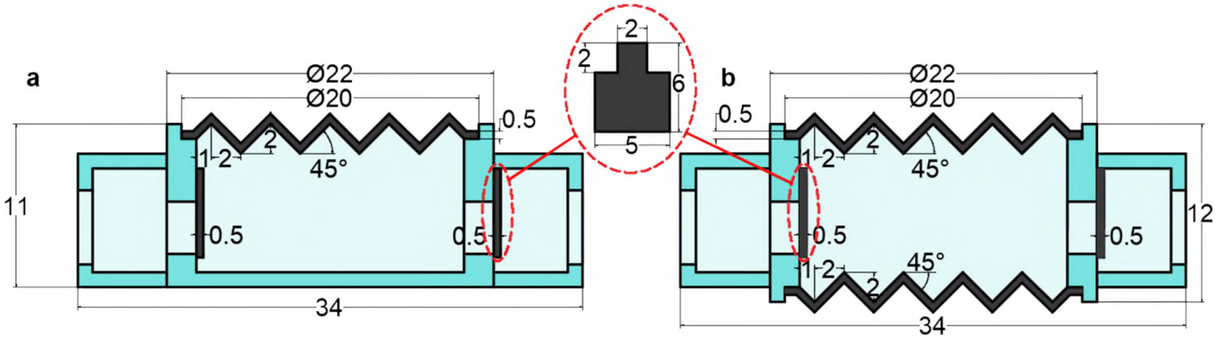


Supplementary Figure 9. **The size information of diaphragm pump. a** Single diaphragm pump. **b** double diaphragms pump.

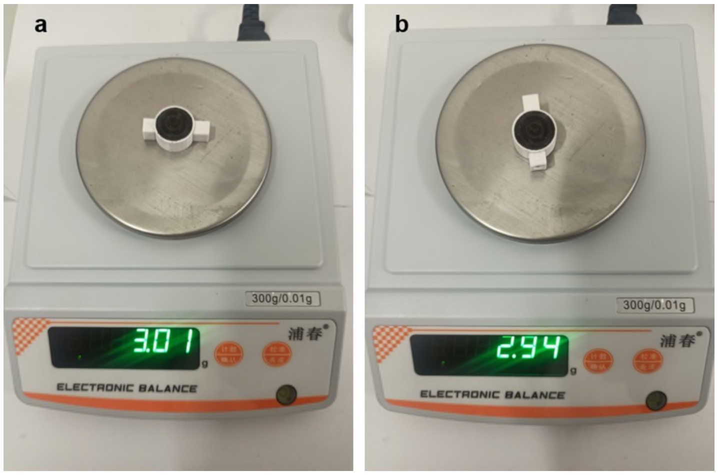


Supplementary Figure 10. **The weight of diaphragm pump. a** Single diaphragm pump. **b** double diaphragms pump.

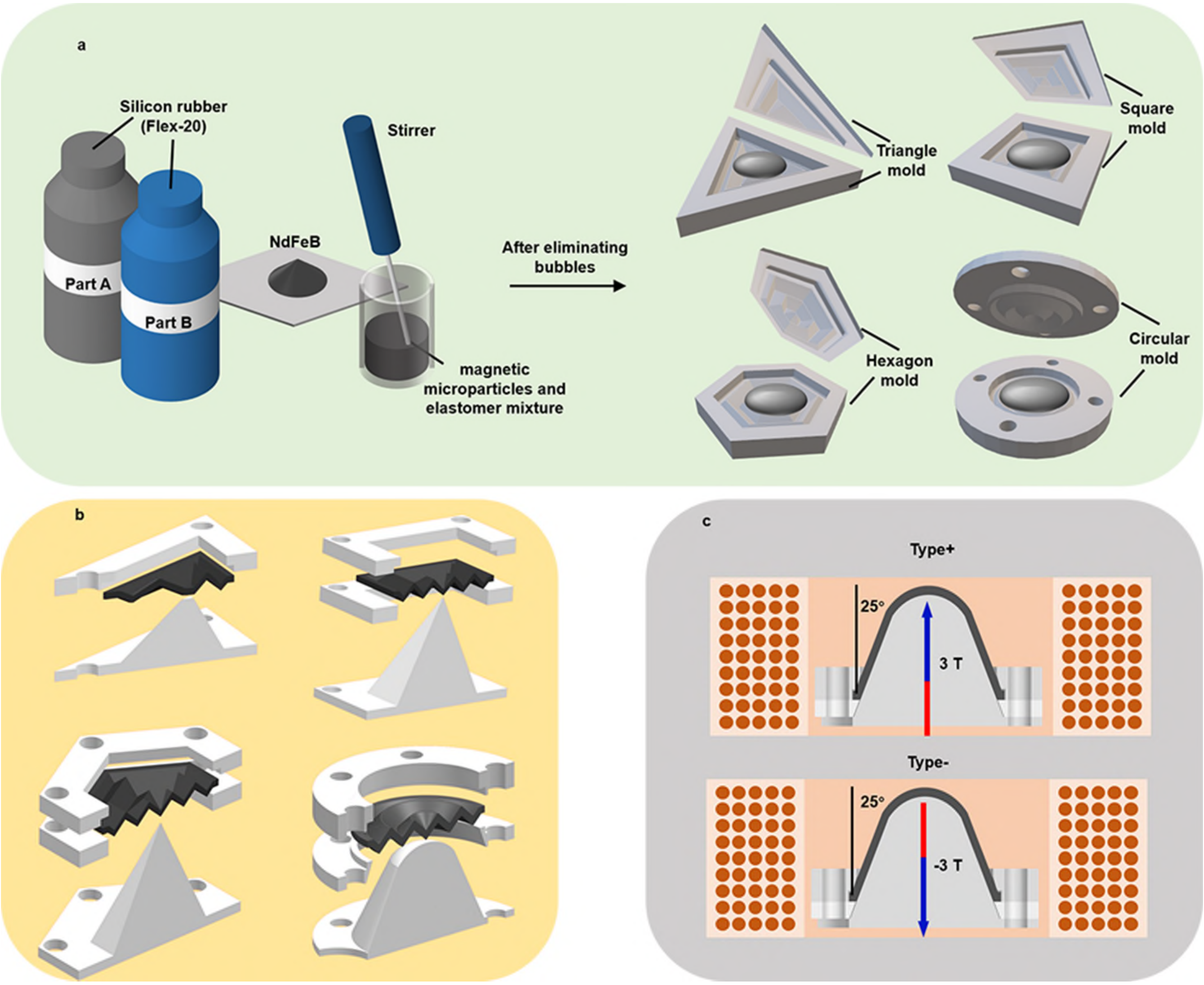


Supplementary Figure 11. **The fabrication process of the folded diaphragm. a** The fabricating process of the folded diaphragm. **b** Four types magnetization model for four shapes of folded diaphragm. **c** The magnetization process of the folded diaphragm.

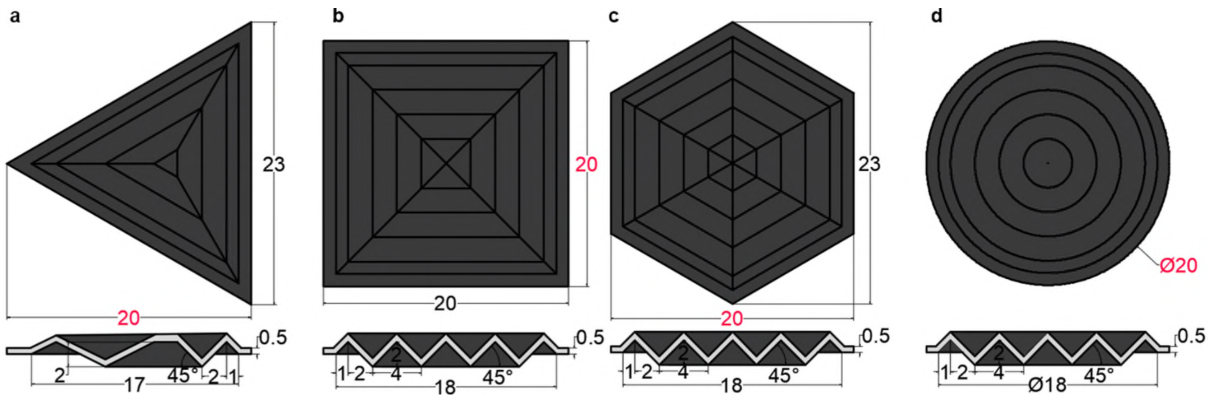


Supplementary Figure 12. **The size information of four configurations. a** Triangle. **b** Square. **c** Hexagon. **d** Circular.

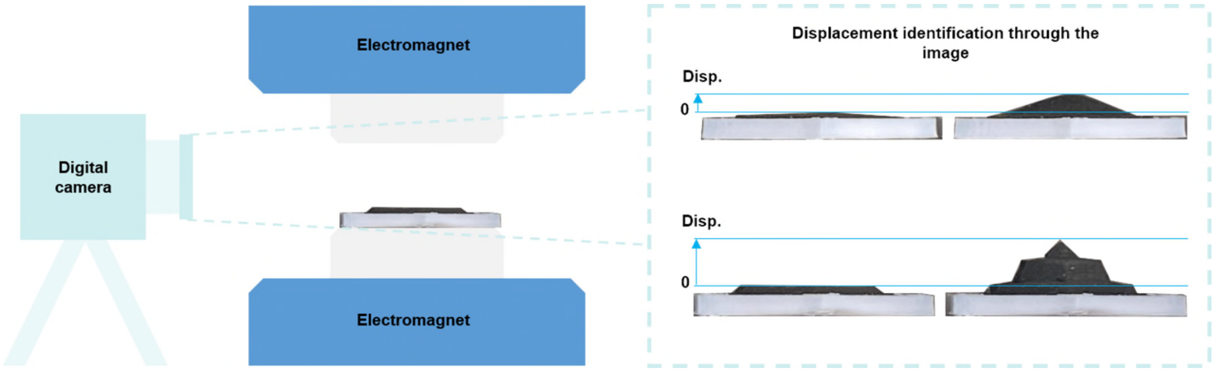


Supplementary Figure 13. **The test platform of deformation property through the image identification technology.**

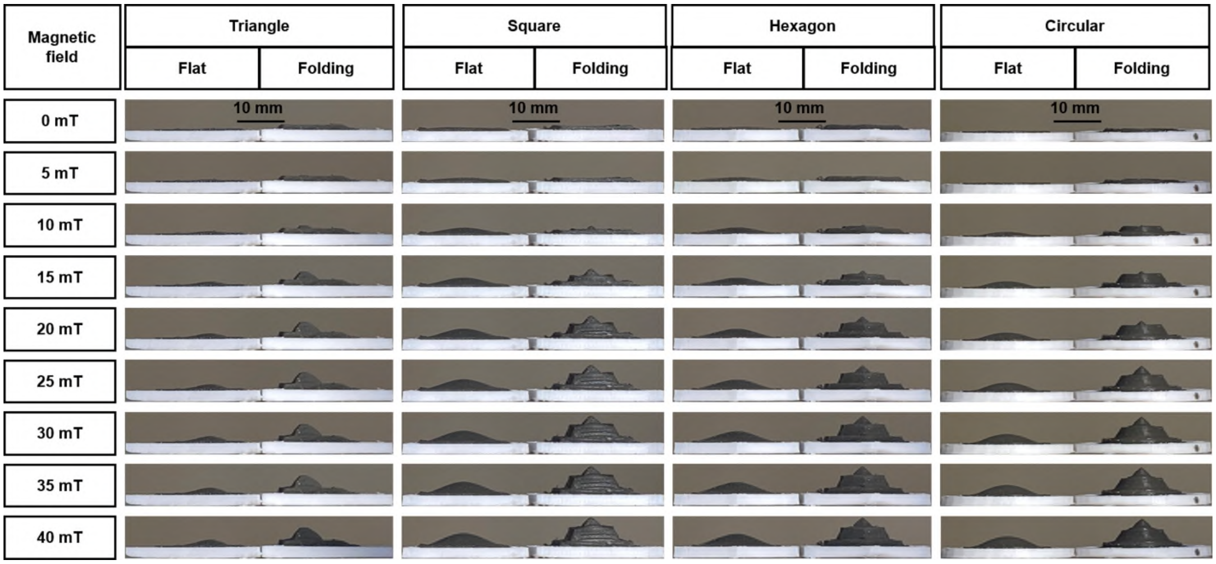


Supplementary Figure 14. **The comparisons of diaphragm deformation of four configurations (triangle, square, hexagon, circular) in front view.**

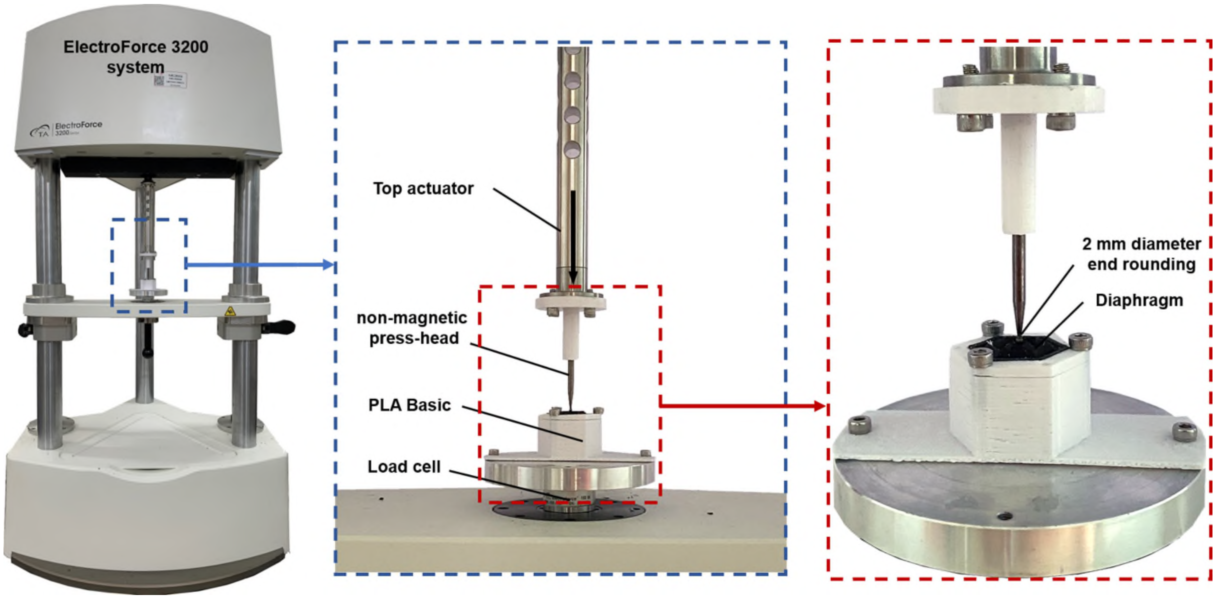


Supplementary Figure 15. **Elastic-resistance force test platform.**

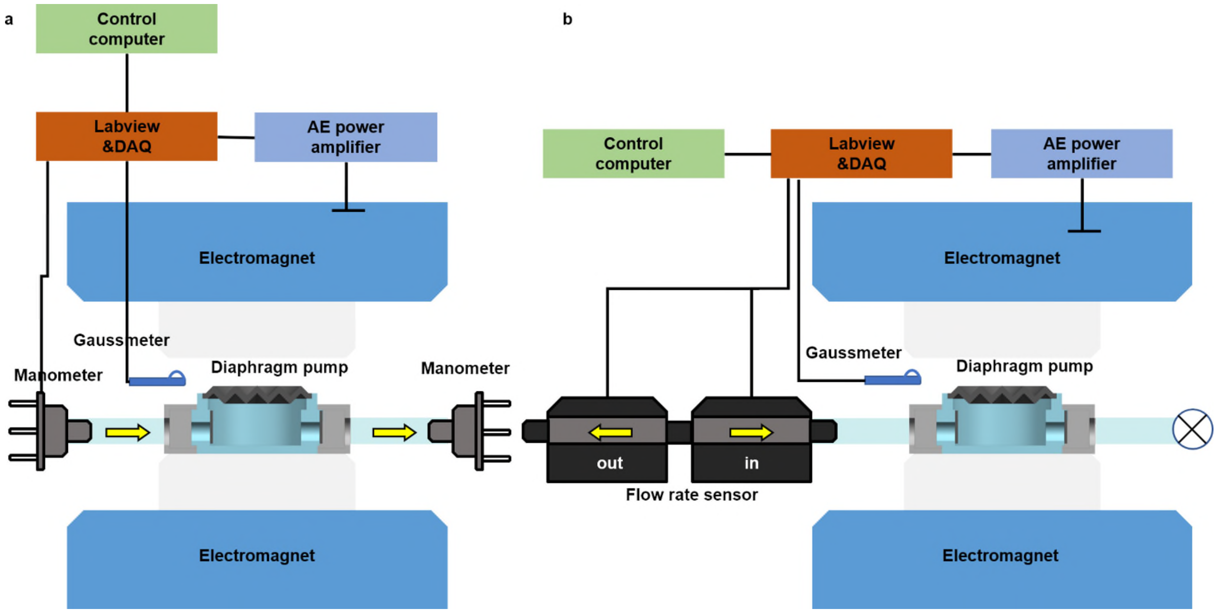


Supplementary Figure 16. **the test platforms of diaphragm pump. a** Pressure test. **b** Ideal flow rate property test.

Supplementary Table 1. **the fabrication conditions of different type of folded diaphragm.**

| Type of sample | thickness | folding degree | number of segments | silicone elastomer | weight ratio (NdFeB to elastomer) | magnetization degree |
|---|---|---|---|---|---|---|
| 1 | 0.5 mm | 45° | 5 | Ecoflex-20 | 1:1 | 65° |
| 2 | 1.0 mm | 45° | 5 | Ecoflex-20 | 1:1 | 65° |
| 3 | 1.5 mm | 45° | 5 | Ecoflex-20 | 1:1 | 65° |
| 4 | 0.5 mm | 35° | 5 | Ecoflex-20 | 1:1 | 65° |
| 5 | 0.5 mm | 55° | 5 | Ecoflex-20 | 1:1 | 65° |
| 6 | 0.5 mm | 45° | 3 | Ecoflex-20 | 1:1 | 65° |
| 7 | 0.5 mm | 45° | 6 | Ecoflex-20 | 1:1 | 65° |
| 8 | 0.5 mm | 45° | 9 | Ecoflex-20 | 1:1 | 65° |
| 9 | 0.5 mm | 45° | 5 | Ecoflex-10 | 1:1 | 65° |
| 10 | 0.5 mm | 45° | 5 | Ecoflex-50 | 1:1 | 65° |
| 11 | 0.5 mm | 45° | 5 | Ecoflex-20 | 2:3 | 65° |
| 12 | 0.5 mm | 45° | 5 | Ecoflex-20 | 3:2 | 65° |
| 13 | 0.5 mm | 45° | 5 | Ecoflex-20 | 1:1 | 45° |
| 14 | 0.5 mm | 45° | 5 | Ecoflex-20 | 1:1 | 55° |

Supplementary Table 2. **The average values and standard deviation of the maximum pressure of single diaphragm pump under different magnetic field.**

| Magnetic field amplitude (mT) | Pump-in pressure (kPa) | | Pump-out pressure (kPa) | |
|---|---|---|---|---|
| | Average value | Standard deviation | Average value | Standard deviation |
| 0 | 0.000 | 0.000 | 0.000 | 0.000 |
| 5 | -0.074 | 0.005 | 0.078 | 0.005 |
| 10 | -0.135 | 0.008 | 0.135 | 0.004 |
| 15 | -0.191 | 0.009 | 0.195 | 0.008 |
| 20 | -0.249 | 0.008 | 0.253 | 0.005 |
| 30 | -0.351 | 0.002 | 0.358 | 0.007 |
| 40 | -0.496 | 0.010 | 0.501 | 0.002 |

Supplementary Table 3. **The average values and standard deviation of the cumulative flow rate of single diaphragm pump under different magnetic field amplitudes and frequencies.**

| Magnetic field amplitude (mT) | Flow rate (mL $min^{-1}$) | | | | | | | |
|---|---|---|---|---|---|---|---|---|
| | 1 Hz | | 2 Hz | | 5 Hz | | 10 Hz | |
| | Average value | Standard deviation | Average value | Standard deviation | Average value | Standard deviation | Average value | Standard deviation |
| 5 | 4.6 | 0.1 | 10.1 | 0.2 | 19.4 | 0.5 | 41.6 | 1.0 |
| 10 | 10.9 | 0.4 | 19.9 | 0.7 | 40.7 | 0.7 | 84.4 | 0.7 |
| 15 | 17.8 | 0.2 | 36.7 | 0.5 | 72.4 | 1.0 | 129.5 | 1.7 |
| 20 | 24.0 | 0.6 | 45.2 | 0.5 | 96.8 | 0.8 | 178.2 | 2.6 |
| 30 | 35.1 | 0.9 | 63.5 | 0.8 | 141.9 | 0.7 | | |
| 40 | 43.7 | 0.6 | 86.7 | 1.2 | | | | |

Supplementary Table 4. **The average values and standard deviation of the cumulative flow rate of double diaphragm pump under different magnetic field amplitudes and frequencies.**

| Magnetic field amplitude (mT) | Flow rate (mL $min^{-1}$) | | | | | | | |
|---|---|---|---|---|---|---|---|---|
| | 1 Hz | | 2 Hz | | 5 Hz | | 10 Hz | |
| | Average value | Standard deviation | Average value | Standard deviation | Average value | Standard deviation | Average value | Standard deviation |
| 5 | 16.1 | 0.2 | 29.6 | 0.2 | 56.4 | 1.0 | 103.6 | 2.2 |
| 10 | 32.1 | 0.2 | 58.2 | 2.1 | 117.4 | 3.4 | 178.1 | 3.5 |
| 15 | 47.6 | 1.3 | 85.0 | 1.4 | 180.5 | 2.2 | | |
| 20 | 60.4 | 0.6 | 113.6 | 0.7 | | | | |
| 30 | 75.9 | 1.0 | 145.1 | 1.0 | | | | |

## Supplementary Reference


[1] Kim, Y. & Zhao, X. Magnetic soft materials and robots. *Chem. Rev.* **122**, 5317-5364 (2022).

[2] Kim, J., Chung, S. E., Choi, S. E., Lee, H., Kim, J. & Kwon, S. Programming Magnetic Anisotropy in Polymeric Microactuators. *Nat. Mater.* **10**, 747–752 (2011).

[3] Hu, W., Lum, G. Z., Mastrangeli, M. & Sitti, M. Small-scale soft-bodied robot with multimodal locomotion. *Nature* 554, 81-85(2018).
