# Supplementary material for: Bio-inspired magnetic-driven folded diaphragm for biomimetic robot
Source: Nat Commun. 2023 Jan 11;14:163. doi: 10.1038/s41467-023-35905-6 (PMC9834404; doi:10.1038/s41467-023-35905-6)
Supplement: Supplementary file 3 — Description of Additional Supplementary Files [file 41467_2023_35905_MOESM3_ESM.pdf]

Description of Additional Supplementary Files

Supplementary Movie 1 Unidirectional fluid transfer function of diaphragm pump

Supplementary Movie 2 The crawling, climbing, turning and over triangular slop locomotion of biomimetic earthworm robot

Supplementary Movie 3 The effect of the magnetic field conditions on the performance of crawling robot

Supplementary Movie 4 Horizontal crawling of biomimetic earthworm robot in the solenoid magnetic field generator

Supplementary Movie 5 Water jet phenomenon of biomimetic squid robot

Supplementary Movie 6 Snorkeling, diving and horizontal swimming of biomimetic squid robot

Supplementary Movie 7 The magnetization process
